# Out of the Closet, Not Yet Out of the House: Gay Men’s Experiences of Homonegativity and Internalized Homonegativity

**DOI:** 10.3390/healthcare9111479

**Published:** 2021-10-30

**Authors:** Jack Thepsourinthone, Tinashe Dune, Pranee Liamputtong, Amit Arora

**Affiliations:** 1 Campbelltown Campus, School of Health Sciences, Western Sydney University, Locked Bag 1797, Penrith, NSW 2751, Australia; t.dune@westernsydney.edu.au (T.D.); a.arora@westernsydney.edu.au (A.A.); 2 Campbelltown Campus, Translational Health Research Institute, Western Sydney University, Locked Bag 1797, Penrith, NSW 2751, Australia; 3 College of Health Sciences, VinUniversity, Gia Lam District, Hanoi 100000, Vietnam; pranee.l@vinuni.edu.vn; 4 Health Equity Laboratory, Campbelltown, NSW 2560, Australia; 5 Oral Health Services, Sydney Local Health District and Sydney Dental Hospital, NSW Health, Surry Hills, NSW 2010, Australia; 6 Clinical School Child and Adolescent Health, The Children’s Hospital at Westmead Clinical School, Faculty of Medicine and Health, The University of Sydney, Westmead, NSW 2145, Australia

**Keywords:** homonegativity, LGBT, gender norms, internalized homonegativity

## Abstract

This paper explores how Australian gay men experience gender and sexuality in relation to heteronormative gender norms, specifically masculinity. A sample of 32 gay men 22–72 years of age participated in an online interview, using a videoconferencing software, on masculinity and homosexuality. Thematic analyses revealed that gay men experience gender and sexuality-related strain across all levels of their socioecological environment through social regulation, homophobic discrimination/harassment, and anti-effeminacy prejudice. The gay men expressed feelings of self-loathing, shame, internalized homonegativity, and isolation as a result. In examining interactions at each level of the socioecological environment, future research and practice may gain understanding in the social phenomena and how to ameliorate such strain.

## 1. Introduction

Beginning from the microsystem, family and close social networks, and extending to the macrosystem, broader social structures and ideologies, the socioecological environment of an individual includes a complex network of formal and informal structures which progressively exist and take shape according to the individuals inhabiting them [1,2,3,4]. Minority stress theory argues that lesbian, gay, bisexual, transgender, and/or intersex (LGBTI) individuals experience chronic stress as a result of the homonegative and heterosexist social environments which they inhabit [5]. Within a gay man’s environment, for example, heteronormative ideals play a noticeable role in the rewarding and stigmatization of traditionally gendered behavior, masculine and effeminate behavior, respectively [6]. Consequently, gay men are socialized to experience negative attitudes towards their own sexuality, internalized homonegativity [7]. Gay men often, for example, experience higher degrees of negative attitudes, abuse, and extreme states of mind (e.g., suicide and homicide) as a result of internalized homonegativity compared with lesbian women [8,9,10,11].

Gender expression encompasses an individual’s conformity (or transgression) of societal gender norms, masculinity and femininity, where in traditional heteronormative and western societies, men are expected to be aggressive, brave, and stoic while women are expected to be emotive, passive, and sentimental [12,13]. Additionally, Bradley [14] explicates that heteronormative masculinity distinguishes itself through the exclusion and oppression of its outgroup actors, women and gay men, who threaten its very essence. However, unlike women who may adopt more masculine traits (e.g., butch, tomboy) without much hostility, men who adopt more feminine traits tend to experience derision from strangers, friends, and family members, notably fathers [14]. Despite flexibility in acceptance of gender expression within most contemporary western society, dichotomization between feminine and masculine gender norms continues to exist [15,16]. Furthermore, these hegemonic norms continue to play a prominent role within gay men’s lives [17].

### 1.1. Being a Gay Man: Literature Review

In the lack of behavioral and verbal cues to signify an individual’s sexual orientation, gender expression is often used as a determinate [12]. Extant literature maintains that gay men are often portrayed as being more effeminate than their heterosexual counterparts, affecting gay men’s perceptions of their own gender and sexual identities [6,18,19]. Phrases such as “that is so gay” or “no homo” are often used as a form of social regulation to deter unscripted expressions of masculinity [20]. However, not only do phrases such as these reflect society’s perceptions of homosexuality but also, they reflect heterosexist ideals. Internalized homonegativity has been noted to relate to depression, poor wellbeing and quality of life, sexual discrimination, shame, body dissatisfaction, eating disorders, and suicidal ideation results in more extreme and unbearable states of mind in men than in women [11,21,22,23,24,25,26]. Phrases such as “I am a man, therefore I may not love a man” [27] and “you can’t be a man and be gay” [18] are common concepts that gay men are regularly confronted with. Scholars posit that these types of homophobic and anti-effeminate sentiments are perpetuated cyclically by victims of gender/sexuality harassment [28].

The strain gay men may experience in their efforts to be as heteronormatively masculine as possible is perpetuated by prejudice and discrimination in all areas of life that serve to govern socially acceptable expressions of masculinity. Minority stress theory argues that people of minority groups are at risk of negative physical and mental health disparities as a result of such stigma and discrimination [29]. Masculine identity and behaviors of overcompensation are posited to be experienced differently among gay men compared with straight men [30]. For example, gay men who are overly concerned with gender norms and masculine body ideals are argued to be compensating for their feelings of internalized homonegativity and inferiority [6,31,32,33]. Additionally, gay men who do not fulfill their own and society’s expectations/ideals of masculinity experience greater psychological distress [17]. Individuals who have experienced harassment due to childhood gender non-conformity are more likely to experience later adult life body shame and bear anti-effeminacy prejudice towards others [28,34]. This is evident in discriminative social practices on classifieds and dating applications which exclude effeminate men [31,35,36,37]. As a result, gay men who have internalized heteronormative masculinity and the strict rules therein participate in policing other gay men, as well as themselves through compensatory behavior, as a means of minimizing gay men’s effeminacy stereotypes [28]. It is further argued that the discrimination between straight-acting and effeminate gay men, particularly within personal advertisements, normalizes, and even glorifies, this divisive social practice. These dynamics thus perpetuate heteronormative masculinity, (hyper)masculine gender norms, and further contributes to gender-related strain and internalized homonegativity.

### 1.2. The Australian Social Environment

The socioecological environment of non-heteronormative and, particularly, sexual minority groups, within a western context, is argued to be a rapidly evolving environment [38]. However, it was only over the last century that more positive attitudes emerged; in 1957, Evelyn Hooker was credited as the first psychologist to challenge the dominant view of homosexuality being a disorder [39]. Subsequently, in 1973 the Diagnostic Manual of Mental Disorders no longer considered homosexuality as a disorder, followed by the World Health Organization’s International Classification of Diseases in 1990 [40]. Within the Australian context, it was only within the last five years, December, 2017, that Australia passed the Marriage Amendment (Definition and Religious Freedoms) Act 2017 which legally allowed the marriage between same-sex couples [41].

Prior to this, discrimination against sexual minority groups was common, with arguments of gay relationships being unnatural [41]. Similarly, the months prior to the amendment saw homophobic and transphobic harassment and assault rise to public attention [42]. A transgender woman who was assaulted, for example, expressed: “I was really scared, I don’t feel as safe as I used to” [42].

### 1.3. Theoretical Framework

This study draws on socio-ecological theory [1,2] which helps to provide a multidimensional and holistic view of the interactions and relationships between diverse social factors. Socio-ecological theory assists in identifying constructs, interactions, and experiences between an individual and various social levels. Notably, this theoretical framework allows for an explicit analysis of the social intricacies of gender through each level of an individual’s environment [43].

The socio-ecological environment of a gay man includes a complex network of structures which progressively exist and take shape according to those who traverse through them [1,2,3]. Socio-ecological theory emphasizes the agency of both the individual and the influence of their formal and informal environments [1]:

- The microsystem, family and close social networks;
- The mesosystem, major settings (e.g., school, church, work);
- The exosystem, other social structures that, although do not contain the individual, encompass their immediate setting, and;
- The macrosystem, broader social structures and ideologies.

**Figure 1:**
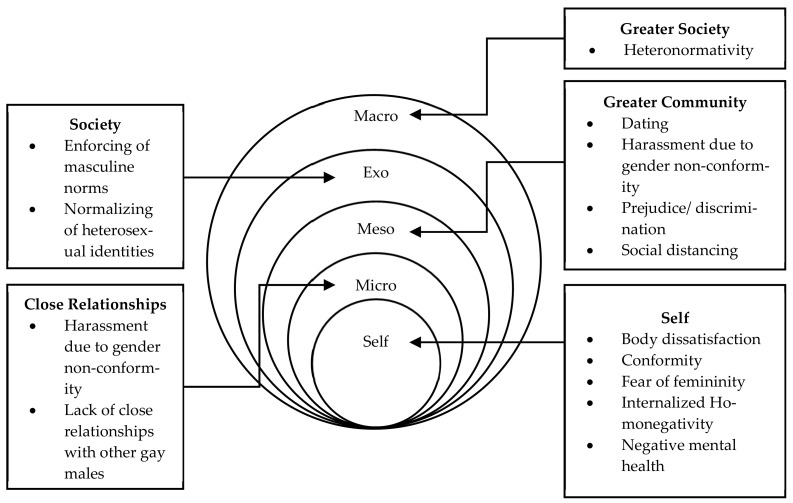
Socio-ecological map of an Australian gay man [4].

Based on extant literature [4], Figure 1 depicts a conceptual model highlighting the typical ecological environment of an Australian gay man whereby heteronormative masculinity pervades and influences various areas of a gay man’s environment. Starting from the macro level, heteronormative gender and sexuality ideals exert a cyclical influence over how gay men perceive themselves, others, and the world through interactions between the individual, their friends and family (microsystem), and strangers and colleagues (mesosystem) [18,28,31,35,36,37,44]. This paper seeks to explore and identify factors within Australian gay men’s socioecological environment that influence how gender and sexual identity and expression are experienced in the context of heteronormativity.

The role of heteronormative masculinity within a gay man’s life should be examined as a complete system of variables contributing to and manufacturing internalized experiences of homonegativity [4]. As such, this study aims to examine these issues and various factors more closely, with particular emphasis on their interconnections.

### 1.4. Present Study

This paper is a part of a larger body of research exploring masculinity and internalized homonegativity amongst gay men. Limited research explicitly focuses on masculinity and internalized homonegativity [4,25,45,46] with very few using qualitative methods [22]. The study, therefore, aims to qualitatively explore this under-examined area, focusing on men’s lived experiences of internalized homonegativity, the factors which contribute to their experiences, and the impact it may have on their health and wellbeing.

Our study asks: how do heteronormative ideals of gender and sexuality impact Australian gay men’s experiences? It is anticipated that the findings will assist in identifying the underlying issues surrounding internalized homonegativity (e.g., gender norms) and epistemological gaps for further exploration. This paper is one in a series of forthcoming papers exploring masculinity and internalized homonegativity amongst gay men.

## 2. Materials and Methods

### 2.1. Recruitment and Participant Demographics

This paper is part of a larger body of research, titled “It’s a Man’s World”, exploring masculinity and internalized homonegativity amongst gay men. Participants were recruited into the “It’s a Man’s World” study via advertisements through LGBTI networks (e.g., LGBTI Alliance of Australia, and Queensland Aids Council), social media (e.g., Facebook, Twitter, and Instagram), dating applications (e.g., Grindr), flyers placed across Western Sydney University campuses, and word of mouth. Advertisements included the researcher’s contact details in order to express interest in the study. Participants from the “It’s a Man’s World” study were provided the option to also express interest in the current research and provide their contact details. A pool of 253 individuals self-identifying as gay men were contacted through email after expressing interest and, from which, only 32 individuals followed up by arranging an interview. No interviews were cancelled or turned down and no participants withdrew from the study.

Using a sample of 32 self-identified gay men 22–72 years of age (M = 34.34, SD = 12.94, median = 30) living in Australia (NSW = 90.63%, QLD = 3.13%, VIC = 3.13%, WA = 3.13%), online interviews focusing on masculinity and homosexuality were conducted between March and July 2017, several months prior to the legalization of same-sex marriage in Australia and during the period in which discussion and contention was high. Among the sample, 3.13% identified as Aboriginal, 6.25% as East Asian, 6.25% as Southeast Asian, and the remainder as Caucasian (85.38%). Additionally, most of the sample identified with No Religion (68.75%), followed by those identifying as Christian (18.75%), Buddhist (6.25%), and Other (6.25%). Gay men are argued to be most adversely affected by heteronormative gender norms and prone to resultant health and wellbeing complications compared with lesbian women [6,25,47]. Therefore, the study’s aim and scope focused solely on gay men and individuals of other genders and sexualities were excluded (e.g., transgender, bisexual, etc.). Each interviewee received a $30 digital gift card as compensation for their time and inconvenience.

### 2.2. Research Design and Interview Guide

Few studies have examined masculinity and internalized homonegativity qualitatively [22]. This study, therefore, intended to explore an under-examined methodology within the field. With considerations of previous studies [18], semi-structured interviews were utilized. The interviews focused on men’s lived experiences of internalized homonegativity and masculinity, with discussions focused on: experiences of childhood harassment due to gender non-conformity, notions of homosexuality as feminine, pressure to be straight-acting/masculine, reactions to gender non-conformity (e.g., anti-femme), and homonegativity. Table 1 displays the interview guide used.

Interviews were facilitated via the Zoom online videoconferencing software which needed to be installed by participants prior to the interview. This software was utilized for two reasons: (a) it enabled the audio-recording of interviews without additional software or equipment and (b) it did not require the researcher to add participants to their contacts, a common requirement in other online conferencing and social media. This allowed for the inclusion of samples from underrepresented, geographically and/or socially isolated populations, and individuals who are unable or prefer not to attend in person [48,49,50]. The gay men, for example, may not wish for their identities to be disclosed and online environments may allow for such populations to participate in research with lower risks to their anonymity [48]. A close-ended self-administered demographics questionnaire was also utilized to ascertain participant’s background information, age, gender, ethnicity, religion, post code, and from what device were they accessing in order to participate (e.g., laptop, phone, or tablet).

### 2.3. Procedure

Following initial contact, the first author (J.T.) forwarded details of the study to the participants, including a participant information sheet, a participant consent form, instructions on how to install and use Zoom, and the time of the scheduled interview. On the day of the interview, participants were required to click on the link included in the email correspondence which automatically prompts the participant to agree to installation of the software. Upon completion of the software installation, Zoom automatically opens the appropriate videoconference session. The researcher then enabled audio-recording on Zoom once consent was provided and commenced a semi-structured interview using the interview guide to facilitate conversation with the participant. All interviews were facilitated by J.T.

### 2.4. Data Analysis

Following data collection, interviews were transcribed verbatim and uploaded into Quirkos. Quirkos is a visually intuitive data management software that assists researchers in the coding and analyses of qualitative data [51]. Quirkos assisted in organizing topical responses and emergent substantive categories. A thematic analysis was used to analyze the data. This was conducted by identifying codes, patterns, and substantive categories within participants’ accounts in relation to the study’s aims [52]. The coding was conducted by J.T. and emerging themes were discussed by all authors (J.T., T.D., P.L., and A.A.).

## 3. Results

To maintain participant anonymity, pseudonyms were assigned to participants where direct quotes were used. Several themes and subthemes emerged from the gay men’s stories, namely: Gay Men and Homonegativity, Gay Men and Identity, and Personal Responses to Homonegativity. Table 2 presents these themes and subthemes and depicts examples of participants’ lived experiences according to each subtheme. The theme of Gay Men and Homonegativity relates to gay men’s experiences of homonegativity within various levels of their socioecological environment. Similarly, the theme of Gay Men and Identity encompasses gay men’s experiences of gender and/or sexual identity in relation to their environment. Lastly, Personal Responses to Homonegativity draws on how gay men relate to their experiences of homonegativity.

### 3.1. Gay Men and Homonegativity

The gay men were asked to recount experiences of homonegativity. In telling their stories, gay men described experiences where both known (e.g., friends and family) and unknown (strangers) individuals within their micro and mesosystems were perpetrators of homonegativity.

#### 3.1.1. Public Homophobic Harassment

The gay men often recounted stories of experiencing homophobic harassment whilst in public. For instance, Tyler (51, Caucasian) recounted “I remember one occasion where I was walking down a street, I had a carload of four guys go past, leaning out the window, calling me a fag”. Tyler’s story highlights an instance of public homophobic harassment from unknown individuals within one’s mesosystem. Aaron (24, Caucasian) shared a similar story: “Someone threatened to knife me. … It was late at night and I was heading home with a friend and some guys came along and thought they would start harassing us. They actually said specifically, ‘When did you choose to be gay?’”

Both Tyler’s and Aaron’s stories share similarities. Despite being from quite different age brackets, they were both harassed by unknown individuals within a public environment. This was a common theme among the stories the gay men shared and, such as these two cases, they were often perpetrated by multiple individuals.

Similarly, Ernest (26, Caucasian) recounts a story of returning to his regional hometown after moving to a capital city: “I think I counted two heterosexist jokes in the space of the first 30 min I arrived. People have just said stuff without even realizing that that may upset me or something. It’s just so ingrained that they don’t even realize that they’re being insensitive or ignorant or whatever”.

Similar to Ernest, other gay men also described experiencing more homonegativity in regional areas compared with larger metropolitan cities. Melvin (30, Caucasian) describes an aversion to regional areas as a result of past homophobic experiences: “I don’t live in rural areas anymore. I now stick to cities because people are much more closed minded in rural areas in my experience”. The heteronormative and homophobic experiences described in participants’ stories highlight the culture embodied within these individuals’ exosystem, notably, differences between regional and metropolitan Australia. When describing changes in acceptance of diverse genders and sexualities, Cooper (26, Caucasian) eloquently stated: “we’re out of the closet now but we’re locked in the bedroom, and I think that we haven’t really yet left the house”. This highlights the limited acceptance of diverse identities within the greater exosystem, which in this context, is Australian society.

#### 3.1.2. Homonegativity from Family/Friends

Additionally, homonegativity was also experienced and perpetrated from known individuals within gay men’s micro and mesosystems. Finn (33, Caucasian) recounts an experience whereby his friends reacted negatively to his gender presentation: “his exact words were, ‘Finn, I knew you told me you were gay, but I didn’t realize you were going to go full blown poofter’. I remember it so vividly. It was shit”. Additionally, Ernest (26, Caucasian) stated: “it’s hard to go, ‘My friends really helped me’, when they kind of embody some of those things”.

Parents, notably fathers, and other family members were also often described as perpetrators of homophobic remarks. Cooper (26, Caucasian) shared this story: “I remember vividly, my dad was driving me to a high school swimming carnival. It was Year 7 or Year 8 or something like that. He basically said to me ‘Dude, you’re not a cat. Cut your goddamn fingernails’. I was really embarrassed … there’s an undertone there. It’s dad saying ‘Cut your fingernails because they’re too long’ but what he’s not saying in words … ‘Stop looking so gay’. … I think my mum has probably said ‘Those jeans are too tight’ or comments about hair”.

Cooper’s story highlights the subtle nuances in homophobic experiences and their ability to become ingrained in individual experiences of internalized homonegativity. A commonality in Cooper and many other participants’ stories is that these experiences can often be recalled vividly and many years after they occurred. This demonstrates the effect interactions with others within one’s microsystem have longitudinally.

Furthermore, the gay men often expressed experiencing negative affects when receiving homonegativity from others within their microsystem. For example, “when my parents found out that I was gay … my parents didn’t speak to me for a year. … That was hard. That was really hard. … It made me feel rejected and unloved” (Isaac, 50, Caucasian). Similarly, Harry (32, Caucasian), expressed: “I want my brother to be proud of me and I want to feel love from my family”. He further explained: “probably my brother was the most impacting one. … More than anyone pegging fruit or yelling things [at me] … that doesn’t matter. Having him say that to me. I was like that was probably the roughest I’ve had”. The gay men often stated that homonegativity expressed from people within their microsystem were more impactful and negative than homonegativity expressed from individuals in other systems of their socioecological environment.

### 3.2. Gay Men and Identity

Gay men were asked to share experiences of self-identity in relation to their sexual and/or gender identities. Their stories often described experiences whereby pressure and regulation of heteronormative ideals were exerted from within their micro, meso, exo, and macrosystems.

#### 3.2.1. Social Regulation

Participants described experiences of homonegativity and heteronormative regulation of individuals’ gender and sexual expressions. When asked to recount previous homophobic and heteronormative experiences, Ernest (26, Caucasian) described an overarching account of the social regulation enforced throughout his life: “We grow up in a world where from the minute a child is born, they’re told that they must behave in a certain way and, if they step outside of those rules, they’re punished. From such an early age, they’re told non-normative genders, non-normative sexualities are bad things”.

Additionally, the gay men who identified as masculine often expressed an incongruence between their gender and sexual identities. Participants expressed receiving pressure to enact stereotypical and caricaturized presentations of gayness (effeminacy) and masculinity from their micro (e.g., family, friends), meso (e.g., colleagues), and exosystems (e.g., film, media). For example: “Gay men in film and television is purely the high camp version, effeminate version of gay men. So therefore, if I didn’t identify with that but I felt attracted to guys there’s a serious disjuncture going on” (Xavier, 32, Caucasian). When experiencing this incongruence, gay men often expressed feelings of self-loathing, isolation, and confusion. For example: “That expectation in a way that society sort of projects okay, you’re gay, so you’re probably going to be more [effeminate]. … Do I hang out with my friends who are all girls and go shopping, or do I go to the gym by myself and be lonely” (Cooper, 26, Caucasian).

Due to the heteronormative pressures exerted from various systems of a gay man’s socioecological environment, gay men experience various states of self, identity incongruence, pressure to conform, self-loathing, isolation, confusion, and internalized homonegativity, for instance.

#### 3.2.2. Regulation from Other Gay Men

When asked about how others in their micro and mesosystem react to effeminate behavior and presentation, the gay men tended to describe receiving negative reactions from other men, notably other gay men: “it’s sometimes considered more negatively, often more by gay guys … because of the whole no femmes, masc tops, all that bullshit” (Finn, 33, Caucasian). They continued to highlight that: “with straight people, it’s not so much a thing. … some guys might find it a little bit confusing and off-putting, but I feel like gay guys are kind of worse about it than a lot of other people”.

Similarly, the gay men reported experiencing discrimination and segregation from other gay men who identify as straight acting; “Rather than pointing and calling names… now we simply exclude them. You’re not behaving straight enough for me and therefore I’m going to exclude you out and I only want to meet straight acting men. It’s not portrayed as a personal preference; it’s portrayed as somehow being better than the alternative” (Tyler, 51, Caucasian).

A hierarchy and valuation of masculinity was often mentioned when describing straight-acting culture, that is, passing as heterosexual was coveted and being noticeably gay was shunned.

### 3.3. Personal Responses to Homonegativity

#### 3.3.1. Fear and Anxiety

The gay men often described experiencing fear or anxiety as a result of both experienced and anticipated homophobic harassment. Thomas (72, Aboriginal) shared his story of how his experiences of homonegativity affected how he has lived his life: “I didn’t do any of those sorts of things. I think perhaps that was a fear. There was a fear that I’d lose my job as a schoolteacher. … some people may not accept gay people as being normal and, therefore, will not allow them to do certain jobs, like some churches and organizations who have this sort of attitude”.

Another commonality among gay men’s stories of homonegativity was that they did not perceive themselves as being visibly gay. For example: “I wasn’t doing anything particularly gay” (Aaron, 24, Caucasian) and “the only instances where I’ve felt threatened or intimidated is when I haven’t been camp or effeminate” (Xavier, 32, Caucasian). The gay men often expressed fear, confusion, and anxiety around how they present themselves in public and how perpetrators discriminate them from other individuals: “It wasn’t like I was running around with a rainbow flag over my shoulder. … Did they see me as being someone that they could pick on and make themselves feel good by enhancing their own masculinity, or did they actually identify something in the way I was walking down the street that made me stand out?” (Tyler, 51, Caucasian).

In response to homophobic experiences, the gay men may often present themselves as straight and/or masculine in order to avoid homophobic harassment, which is known as passing. However, this form of self-regulation is described to be a consumption of one’s internal resources: “passing is something that requires a lot of energy” (Aaron, 24, Caucasian).

#### 3.3.2. Internalized Homonegativity

When asked about their experiences of homonegativity and heteronormative ideals, participants often described feelings of shame, self-loathing, isolation, and internalized homonegativity. Ernest (26, Caucasian) explained: “Being a faggot was the worst thing you could be, really”. Similarly: “I went through a certain period probably when I was about 17/18 of quite significant self-loathing around my sexuality” (Xavier, 32, Caucasian).

These gay men’s stories highlight the pervasive nature of heterosexism and homonegativity and emphasizes the internalization of such ideals within a gay man’s psyche. This was articulated in other participants stories, including: “queer people still embody the same attitudes of heterosexism and shame” (Finn, 33, Caucasian) and “you can drown in [self-loathing] all day” (Xavier, 32, Caucasian).

## 4. Discussion

**Figure 2:**
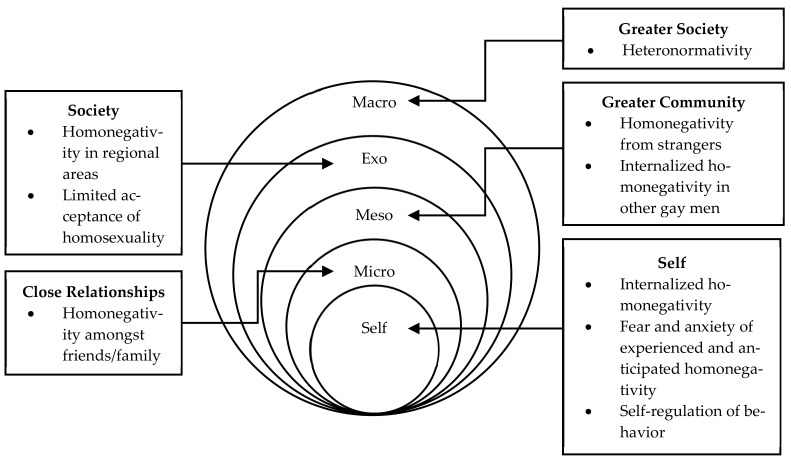
Socio-ecological map of a gay man.

This paper examined gay men’s stories of being both gay and a man. From their experiences of homonegativity and internalized homonegativity, we identified areas within their socioecological environments which exert influence over their sense of gender, sexuality, experiences of gender norms, and the impact it may have on their health and wellbeing. Figure 2 provides an overview of the results in accordance to their respective level on the socio-ecological model.

In examining gay men’s stories, it was ascertained that gay men experience gender and sexuality strain from all levels of their socioecological environment. Beginning from their family/friends in their microsystem; colleagues and unknown individuals in their mesosystem; film, media, and geo-specific cultures (e.g., regional and metropolitan) in their exosystem; and broad societal ideals and norms within the macrosystem. These strains may manifest in the form of social regulation, homophobic discrimination and harassment, and anti-effeminacy prejudice. As such, gay men experience feelings of self-loathing, shame, internalized homonegativity, and isolation. This is similar to previous literature which highlight gay men’s experience of depression, poor wellbeing and quality of life, sexual discrimination, shame, body dissatisfaction, eating disorders, suicidal ideation, and results in more extreme and unbearable states of mind [11,21,22,23,24,25,26]. By examining gay men’s experiences qualitatively and holistically, we identified areas where these strains are experienced. In doing so, interactions between each level of the socioecological environment can be identified where they may conflate and/or coincide with each other.

Gay men within the present study expressed experiences of identity incongruence, self-loathing, and internalized homonegativity. Homonegativity and heterosexism have historically been integral components in the conception and reproduction of hegemonic masculinity [53]. So much so that homosexuality and masculinity are perceived as two separate identities, exclusive from one another [18,27]. As such, it can be maintained that gay men’s constructions of masculinity impose a strain on their self-identity and become unable to fulfill their own perceptions of masculine identity, creating an oxymoron out of the phrase “gay man”. Gay men who do not fulfill their own, as well as society’s, expectations and ideals of masculinity thus experience greater psychological distress [17]. This strain is an alarming issue and should be addressed in clinical practice.

Additionally, Diefendorf and Bridges [53] maintain that although the prevalence of homophobic attitudes and prejudice has decreased over time, enactments of homonegativity continue to be reported. This is evident within the present study whereby gay men reported incidences of homonegativity from strangers, friends, and family within their micro and exosystems. The gay men tended to highlight the vividness of recall and impact of homophobic experiences enacted by members of their family. This is consistent with current literature which emphasize the role of family members (e.g., fathers) on gay men’s experiences of gender and sexuality [14]. Within the Alessi [5] case study, chronic minority stress experienced during an individual’s formative years and enacted by family members and others within an individual’s micro and mesosystems were argued to have lasting effects on later adult life coping and mental health. Furthermore, participants within the present study often cited that it was men who perpetrated acts of homonegativity. Fisher, et al. [54] highlights the higher prevalence of homonegativity and transphobia from men, as opposed to women. As such, the role of male family members and close others on an individual’s experiences may need to be examined when addressing issues of (internalized) homonegativity.

Furthermore, the stories shared by the gay men within this study depict anti-effeminacy and social-regulation of gender expression among other gay men within their micro and mesosystems, notably straight-acting gay men. It is suggested that gay men who conform more to masculine norms, as well as self-identify as straight-acting, possess higher degrees of internalized homonegativity [4,55]. Thepsourinthone, Dune, Liamputtong and Arora [4] argued that gay men high in internalized homonegativity are motivated to maintain a distinct gender identity from other gay men (in this case, femme gay men) through perceptions of masculinity. Additionally, gay men who harbor more negative attitudes toward effeminacy possess more internalized homonegativity and tend to place importance on others’ degree of masculinity [56]. Considering that gay men also receive harassment due to homonegativity and gender expression from others within their micro, meso, and exosystems, it is alarming that other gay men perpetuate similar harassment behavior within their micro and mesosystems. As a minority demographic, this intra-group conflict can be regarded as a distressing social phenomenon. As such, it is recommended that future research and practice aim to examine and alleviate such intra-group conflict.

### Limitations

Limitations of the present study may include the lack of ethnic diversity (mostly Caucasian), age, and sexual identities (only examined gay men). Future studies may wish to examine other non-heteronormative identities, ethnicities, and age groups in order to ascertain the broader spectrum of how gendered norms impact non-heteronormative individuals and whether these impacts are unique to particular identities or are a shared experience.

Additionally, the minority stress model may not be without fault. Bailey [57] provided criticism of the minority stress model such as its over-reliance of self-reported data and neuroticism in individuals’ temperament, the present study’s sample consists of volunteers and may hold self-selection bias. Future studies may wish to examine homonegativity through other models or by employing experimental (as opposed to non-experimental) research designs. A resilience model, accounting for an individual’s ability to persevere and thrive through adversity and significant stressors [29,58], may provide further insight into a healthy and non-fatalistic understanding of gay men’s experiences of homonegativity and internalized homonegativity and may address issues of neuroticism in participants.

## 5. Conclusions

Sparse are current researches explicitly examining masculinity and internalized homonegativity [4,25,45,46] and even sparser are those employing a qualitative approach [22]. We addressed this gap by qualitatively examining Australian gay men’s stories on homonegativity, masculinity, and the interactions between varying socio-ecological systems. It was ascertained that gay men experience gender and sexuality strain from all levels of their socioecological environment which are often experienced in the form of social regulation, homophobic discrimination and harassment, and anti-effeminacy prejudice. As such, gay men experience feelings of self-loathing, shame, internalized homonegativity, and isolation. Our findings contribute to furthering the sociological understanding of LGBTI and men’s health, and we recommend future studies to further explore the topics uncovered within this paper. A wholistic perspective is recommended to understand and examine interacting relations and to adequately address them in practice. In adopting both a minority stress and socioecological approach, a better understanding of the intersectional stressors experienced by gay men may be achieved.

## Figures and Tables

**Table 1:** Interview question guide.

Item No.	Question
1	How would you describe your understanding of society’s perceptions of male homosexuals?
2	It has been an old saying that gay men are typically feminine. What are your thoughts on this?
3a	What is your perception of what it means to be feminine?
3b	What is your perception of what it means to be masculine?
3c	Would you describe yourself as possessing more masculine/feminine characteristics?
4	Have you ever experienced pressure to behave more/less masculine/feminine?
5	Has this perception of homosexuality impacted your experiences growing up?
6	Have you ever experienced anti-feminine reactions from other people or been a witness to such an event?
7	Do you think it is important for men to act masculine?
8	I am about to read to you a few common feelings gay men have expressed in other studies about who they are.
8a	‘You can’t be a man and be gay’. What do you think of when you hear this?
8b	‘You’re less of a man simply because you don’t sleep with women’. What do you think of when you hear this?
8c	Have you ever felt or said anything like this before?
9	Do you ever have negative thoughts/feelings about being gay?
10	In your opinion, what influences gay men to feel negatively about their own queer identity?
11	This research hopes to reduce the stigmatization of what it means to be a gay man. Do you think reducing this stigma can help gay men experience less gender-related strain?

**Table 2:** Summary of emergent themes and subthemes from participants’ stories.

Themes/Subthemes	Example Quote
Gay Men and Homonegativity	
Public Homophobic Harassment	I’ve been walking down the street with my boyfriend and been heckled out at cars and things like that. (Anthony, 23, Caucasian)
Homonegativity from Family/Friends	So, his exact words were ‘Finn, I knew you told me you were gay, but I didn’t realize you were going to go full blown poofter’. I remember it so vividly. It was shit. (Finn, 33, Caucasian)
Gay Men and Identity	
Social Regulation	From such an early age, they’re told non-normative genders, non-normative sexualities are bad things. (Ernest, 26, Caucasian)
Regulation from Other Gay Men	Certain parts of our society experience enough discrimination already without having to receive those sorts of messages from what’s supposed to be a fairly embracing and welcoming community. (Finn, 33, Caucasian)
Personal Responses to Homonegativity	
Fear and Anxiety	I didn’t do any of those sorts of things. I think perhaps that was a fear. There was a fear that I’d lose my job as a schoolteacher. (Thomas, 72, Aboriginal)
Internalized Homonegativity	A lot of queer people would have negative opinions about themselves, often sometimes without even realizing it. (Ernest, 26, Caucasian)

## Data Availability

The data associated with the paper are not publicly available but are available from the corresponding author upon reasonable request.
